# Continuous, Large-Scale, and High Proportion of Bioinspired Phosphogypsum Composites via Reactive Extrusion

**DOI:** 10.3390/ma14195601

**Published:** 2021-09-27

**Authors:** Jingyu Ran, Xiangdong Su, Jiangang Zhang, Jie Zhang, Jiajun Chen, Kun Liu, Zhao Liu, Yi Hu, Liqun Sun, Deyong Jiang

**Affiliations:** 1 Guizhou Phosphogypsum Institute, Guizhou Institute of Technology, Guiyang 550003, China; 20211054@git.edu.cn (J.Z.); 19970210cjj@sina.com (J.C.); liukun51@hotmail.com (K.L.); 2 Key Laboratory of Light Metal Materials Processing of Guizhou Province, Guizhou Institute of Technology, Guiyang 550003, China; zhangjg009@126.com; 3 Guizhou Haobainian Housing Industry Co., Ltd., Guiyang 550000, China; liuzhaohaobainian@126.com; 4 Bijie Yuyuan New Materials Co., Ltd., Bijie 551700, China; tangjiuz@163.com; 5 Guizhou Building Material Quality Supervision Testing Center, Guiyang 550014, China; sunny0309c@126.com (L.S.); jdy1968@126.com (D.J.)

**Keywords:** bio-inspired material, beta-hemihydrate phosphogypsum, reactive extrusion, mechanical strength, softening coefficient

## Abstract

Biological matter evolution provides an idea for the human design and synthesis of new materials. However, biomimetic materials only stay in laboratory-scale models, and their large-scale industrial applications are yet to be realized. Here, inspired by nacre’s architecture, we report a continuous, large-scale method to fabricate phosphogypsum composites by reactive extrusion strategy. After curing for seven days, with more than 50 wt% of beta-hemihydrate phosphogypsum (β-HPG), the compressive strength and softening coefficient were 24.98 MPa and 0.78, increasing by 110.0% and 20.0%, respectively, compared to the pouring method. The results show that the screw extrusion process can improve the mechanical strength and waterproof properties of β-HPG hydration specimens without any special chemical admixtures and cements.

## 1. Introduction

Phosphogypsum (PG) is an industrial by-product generated from the wet-process phosphoric acid, and large amount of PG are discharged as industrial solid waste [1]. The untreated PG occupies many lands and causes serious pollution to the surrounding ecological environment (such as groundwater, atmosphere, soil, etc.). Beta-hemihydrate PG (β-HPG) is obtained from the dehydration of original PG, which contains the main chemical component of calcium sulfate hemihydrate (CaSO_4_∙0.5H_2_O) [2]. However, the mechanical strength, waterproofness, and durability of the β-HPG hydration materials are poor, thus limiting the high proportion of β-HPG in the construction industry.

Over the past few decades, chemical admixtures [3] and special cements [4] have been investigated to improve the performances of gypsum building materials. However, these high-cost treatment methods limit the application of building gypsum on an industrial scale. Much effort has been proposed for controlling the crystallization and morphology of calcium sulfate, such as inorganic additives [5,6,7,8], organic acids [9], surfactants [10,11], and aqueous-organic systems [12], but the as-prepared samples only presented rods, plates, and whiskers. A more interesting microstructure has not yet been reported. As a source of inspiration, biological matter evolution provides an idea for human design and preparation of new materials. These new materials have an almost perfect micro/nanostructure and functions [13,14]. However, due to the high technical cost (e.g., layer by layer deposition [15,16], frozen casting process [17,18], pulsed laser beam [19], etc.), a lot of research works related to biomimetic materials only stay in laboratory-scale models, and the large-scale industrial applications have yet to be realized.

It has been observed that CaSO_4_∙0.5H_2_O is recrystallized into calcium sulfate dihydrate (CaSO_4_∙2H_2_O) during hydration [20]. Given this, we propose that it is the process that allows adjusting and controlling the morphology of the gypsum crystal. As a process intensification technology, extrusion technology has been widely used to blend and process in the polymer [21], pharmaceutical [22], and food industries [23], and in inorganic and organic materials synthesis [24,25]. Extrusion technology has also been applied in the field of building materials, and gradually presents new development characteristics [26,27,28,29,30].

It is worth noting that reactive extrusion was selected in 2019 by IUPAC as one of the ten chemical innovations that will change the world. In the foreseeable future, reactive extrusion as a strengthening process under the action of mechanical force will be a new method for the synthesis of inorganic materials. Therefore, the innovations of this work are as follows: (1) the concept of bioinspired PG composites is firstly proposed, which is in contrast to the “brick and mortar” micro-structure of mature nacre; (2) the process strengthening technology of reactive extrusion is used to fabricate bioinspired structural PG composites, and the influence on the crystal structure of calcium sulfate is discussed; and (3) large-scale bioinspired PG composites have been successfully applied in industrial production.

## 2. Experimental Section

### 2.1. Materials

Ordinary Portland cement type II 42.5R (OPC) and β-HPG were used in all mixtures. To ensure the universality of the experimental results, we selected β-HPG from three places, and the main chemical compositions are shown in Table 1, indicating that β-HPG contains over 85.00 wt% CaSO_4_∙0.5H_2_O (calculated from the contents of SO_3_ and CaO) and small amounts of SiO_2_, P_2_O_5_, Al_2_O_3_, and Fe_2_O_3_. The chemical and bogue composition of the OPC are provided in Table 2. Polycarboxylate superplasticizer, retarder (citric acid) and Hypromellose (BASF AdvancedChemicals Co., Ltd., Shanghai, China) were used to optimize the hydration and molding process of specimens. All reagents and raw materials were commercial.

### 2.2. Preparation of HPG-P Composites

The hydrated β-HPG composites prepared by the pouring method labeled as HPG-P. The mixture proportions for the HPG-P composites are shown in Table 3, and the water slurry (W/S) ratio of the paste was 0.50. According to the GB/T17671-1999 standard [31], the mixtures were molded into a cuboid of 40 mm × 40 mm × 160 mm and compacted on a vibration table. Specimens were molded at 20 ± 2 °C, 60 ± 5% RH for 24 h, then demolded and cured for seven days in the same environment for the tests of mechanical strength.

### 2.3. Preparation of HPG-E Composites

**Scheme 1:**
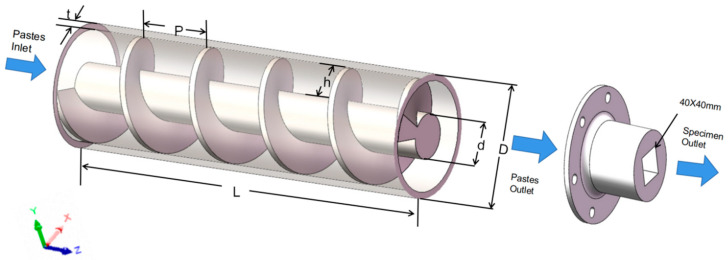
Geometry parameters of the single-screw simulation.

The hydrated β-HPG composites prepared by extrusion labeled as HPG-E (Supplementary Movie S1). The mixture proportions for the HPG-E composites are shown in Table 4, and the W/S ratio of the paste was 0.35. All of the materials entered through the feed port of the screw extruder, and then the materials were extruded through the process of screw rotary extrusion. The mold located at the extrusion port was customized according to the size of the pouring method. The as-prepared specimens size was 40 mm × 40 mm × 160 mm. All the specimens were placed in a standard curing room at ~65% RH and 20 ± 2 °C for 24 h, then cured to seven days at the same environment for the tests of mechanical strength. Scheme 1 illustrates the continuous single-screw extrusion process for fabricating HPG-E composites; the specific parameters of the extruder are listed in Table 5.

### 2.4. Characterization

X-ray diffraction patterns of the as-prepared samples were investigated on an Ultima-IV (M/s. Rigaku Corporation, Tokyo, Japan) with nickel-filtered Cu Kα radiation (λ = 1.54056 Å) and a 2θ value ranging from 2 to 70° at a scanning rate of 0.02°/step. The accelerating voltage and the applied current were 40 kV and 40 mA, respectively. The morphology of the samples was detected by using a FEI Novsa Nano SEM 450 system at an accelerating voltage of 3 kV. X-ray photoelectron spectroscopy (XPS) was performed on a ULVAC PHI Quantera microscope. The specific surface area and pore volumes of samples were measured using a 3H-2000PS1 (Beijing, China) type analyzer by nitrogen adsorption at liquid nitrogen temperature, BET and BJH analysis were used to determine the surface area and pore size distribution of the samples. Raman spectroscopic studies were carried out on a HORIBA JY LabRAM HR Evolution, operating at 532 nm, excitation laser source at 50 mw).

### 2.5. Mechanical Tests

After curing for seven days, the mechanical strength of specimens was measured by a fully automatic test machine (HYE-3000, Cangzhou Zhongjian Precision Instrument Co., Ltd., Hebei, China) following the Chinese standard (GB/T 9776-2008) [32]. Three specimens with a size of 40 mm × 40 mm × 160 mm were prepared for each mix proportion. The loading rates of compressive strength and flexural strength tests were 2400 N/s and 50 N/s, respectively.

### 2.6. Water Absorption and Durability Test

The values of the water absorption as percentages were calculated with the following equation:

(1) $$W_{A(t)}=\frac{W_{t}(Kg)-W_{0}(Kg)}{W_{0}(Kg)}\times 100\%$$

where *W_A_*_(*t*)_ is the water absorption at time *t*, *W*_0_ is the original weight, and *W_t_* is the sample’s weight at a given soaking time *t*.

The softening coefficient (*K*) was implemented to assess the water resistance and weather fastness, calculated by Equation (2). Each group was replicated six times.

(2) $$K=\frac{f(MPa)}{F(MPa)}$$

*K* is the softening coefficient, *f* represents the compressive strength of the specimens soaked in water for seven days, and *F* is the specimen’s compressive strength after curing for seven days.

### 2.7. Radioactivity Test

The radioactivity of the specimens was determined according to the Chinese standard (GB6566-2010) [33]. The internal exposure index (*I_Ra_*) and the external exposure index (*I_r_*) were figured to determine whether as-prepared specimens can be used as construction and building materials. *I_Ra_* and *I_r_* were calculated by Equations (3) and (4), separately.

(3) $$I_{Ra}=\frac{C_{Ra}}{200}$$

(4) $$I_{\gamma }=\frac{C_{Ra}}{370}+\frac{C_{Th}}{200}+\frac{C_{k}}{4200}$$

### 2.8. Simulation of Screw Extrusion

POLYFLOW software (19.2 version) was used to simulate the screw extrusion process. This work aims to analysis the flow condition in the screw channel. The geometry of screw was drawn with the scale of 1:1 by SOLIDWORKS software. Meshing was carried out by using the preprocessor software in the POLYFLOW. Boundary conditions were set based on the momentum and continuity equations [34]. A convergence result analyzed the pressure and velocity solution of the simulation condition. When the physical simulation model was revisited, the following assumptions were made for the flow: (1) ignore the volume force of gravity; (2) the fluid was incompressible viscous fluid; (3) the flow field was steady and isothermal; (4) the wall has no-slip; and (5) the flow was laminar.

Continuity equation:

(5) $$\frac{\partial v_{x}}{\partial x}+\frac{\partial v_{y}}{\partial y}+\frac{\partial v_{z}}{\partial z}=0$$

Momentum equation:

(6) $$\begin{array}{l} \frac{\partial p}{\partial x}=\frac{\partial \tau_{xx}}{\partial x}+\frac{\partial \tau_{yx}}{\partial y}+\frac{\partial \tau_{yx}}{\partial z}\\ \frac{\partial p}{\partial y}=\frac{\partial \tau_{xy}}{\partial x}+\frac{\partial \tau_{yy}}{\partial y}+\frac{\partial \tau_{yz}}{\partial z}\\ \frac{\partial p}{\partial y}=\frac{\partial \tau_{xz}}{\partial x}+\frac{\partial \tau_{yz}}{\partial y}+\frac{\partial \tau_{zz}}{\partial z} \end{array}$$

Shear rate:

(7) γ=π×D×60∗N×h

*γ* is the shear rate in the screw channel. *D* is the screw diameter. *N* is rotation speed and *h* is channel depth.

## 3. Results and Discussion

### 3.1. Mechanical Properties

**Figure 1:**
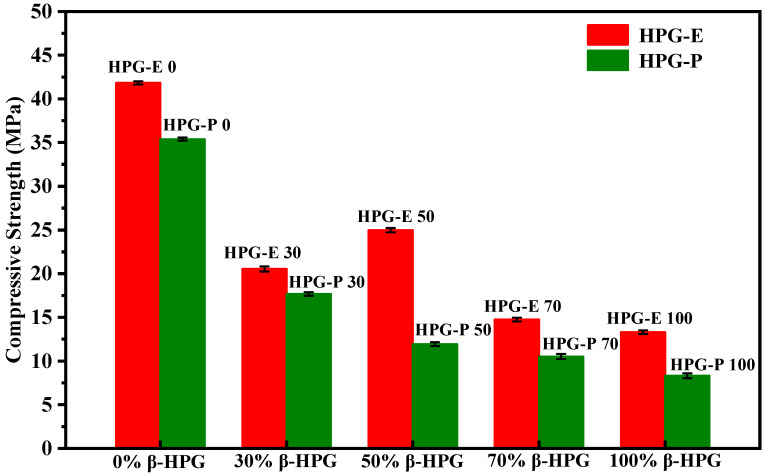
Compressive strength of HPG-P composites and HPG-E composites.

**Figure 2:**
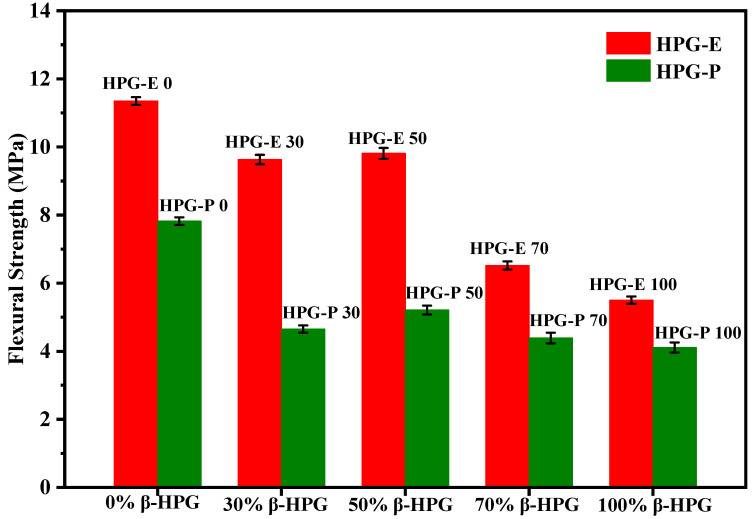
Flexural strength of HPG-P composites and HPG-E composites.

The mechanical strength of the as-prepared specimens is shown in Figure 1 and Figure 2. The error bar is included to illustrate the results of multiple groups of experiments. The HPG-E composites exhibited outstanding compressive strength and flexural strength, superior over the HPG-P composites. The compressive strength of HPG-E composites ranging from 30% to 100% β-HPG was increased by 16.18%, 109.38%, 40.21%, and 59.86% compared to the HPG-P composites. The flexural strength of specimens was also increased by 107.9%, 88.29%, 48.52%, and 24.09%, respectively. When the content of β-HPG was 30% and 50%, the compressive strength of specimens was 20.54 MPa and 24.98 MPa, respectively. However, the compressive strength of specimens began to decline with the further increasing of β-HPG content.

It is worth noting that the mechanical properties of the specimens can be effectively improved by the strengthening effect of screw extrusion. The addition of OPC played an effective role in increasing the mechanical strength of β-HPG hydration products. However, the existence of a large amount of OPC may lead to the formation of ettringite in the later stage. More promisingly, with the aid of screw extrusion, the specimens can have higher mechanical properties with lower OPC content. From the macro point of view, extrusion molding can promote the specimens to be more compact and require low W/S ratio. However, the reason of the great improvement on mechanical properties is not clear from the macroscopic view. In this regard, we speculated that due to the different processing and molding methods, the calcium sulfate crystal changed in the hydration process, making the properties of gypsum changed. Therefore, we carefully observed the microstructure of the gypsum composite.

### 3.2. Microstructure Analysis

**Figure 3:**
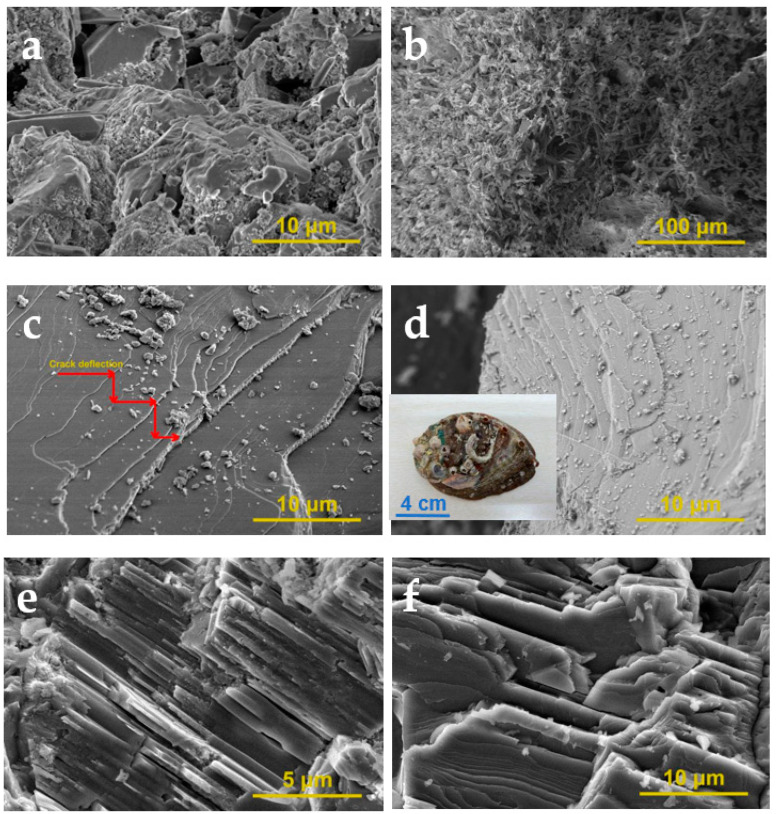
SEM characterization of specimens. (**a**) The cross-section of HPG-P 50; (**b**) the sample obtained by mixing 50% β-HPG and hypromellose by pouring method; (**c**) the cross-section of HPG-E 50; (**d**) the micro-structure of the Haliotis gigantea’s shell (the inset is the shell of Haliotis gigantea); (**e**) the cross-section of HPG-E 30; (**f**) The cross-section of HPG-E 70. (The cross-section of the samples was obtained by quenching in liquid nitrogen.).

As mentioned above, SEM characterization was used to investigate the change of micro-structure affected by reactive extrusion strategy and pouring method, further explaining the strengthening mechanism of screw extrusion process on the properties of β-HPG hydration specimens. Comparing microstructure of the different images shown in Figure 3, it is observed that calcium sulfate dihydrate crystals are interconnected to form the network structure, and the poor water resistance of β-HPG hydration specimens is due to the weakness of contact points of calcium sulfate dihydrate (Figure 3a) [35]. To prove that the micro-structure does not affect by other admixtures, β-HPG and hypromellose were mixed by pouring but the additive only increased the ratio of length to the diameter of the gypsum crystal (Figure 3b). As shown in Figure 3c, it can clearly be seen that the crystal morphology of HPG-E presents a two-dimensional crystal structure, and the embedded hydrated calcium silicate (C-S-H) gels and ettringite increase the number and area of contact points among the calcium sulfate dihydrate crystals. In the section analysis of HPG-E composites, it is found that the micro-structure of HPG-E composites stay the same form of lamellar stacking arrangement with different content of β-HPG (Figure 3e,f). This phenomenon can be attributed to the process strengthening by reactive extrusion. Under external pressure, the nucleation rate of calcium sulfate dihydrate crystals increased and the growth rate decreased, making it easy to obtain special nano-crystals [36]. In addition, many nano-amorphous C-S-H particles were doped in it as the synapses and bridges. This kind of architecture is very similar to the nacre’s microstructure (Figure 3d), thereby improving the mechanical properties of specimens. It is worth mentioning that a tougher mechanism occurring at several length scales was observed in the cross-section of HPG-E composites, and the crack patterns were tortuous; the detailed fracture process is indicated by arrows in Figure 3c. Due to the presence of long-range lamellar architecture, HPG-E composites exhibit more excellent mechanical properties, which provide a useful idea for us to design larger scale high-performance PG building materials.

### 3.3. X-ray Diffraction (XRD) Analysis

**Figure 4:**
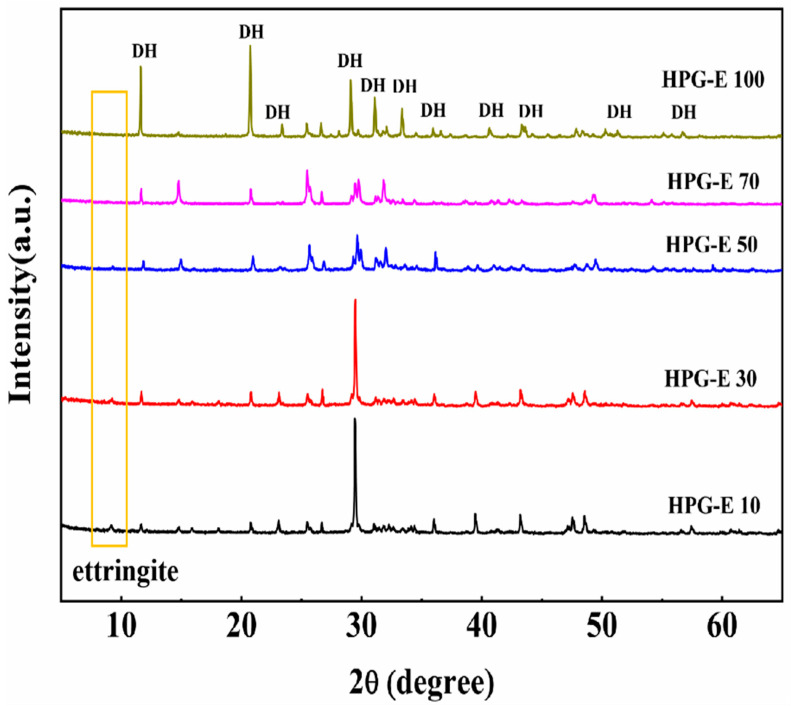
XRD patterns of the as-prepared specimens.

The XRD pattern (Figure 4) shows peaks at 11.69, 20.79, 23.46, 29.19, 31.12,31.2, 32.11, 33.42, 35.96, 36.67, 40.74, 43.37, 43.68, 51.35, and 56.83 corresponding to the (0 2 0), (−1 2 1), (0 4 0), (−1 4 1), (1 2 1), (0 0 2), (−2 1 1), (0 2 2), (2 0 0), (−2 2 2), (−1 5 2), (2 4 0), (−2 5 1), (2 6 0) and (−2 0 4) facets of monoclinic phase CaSO_4_·2H_2_O (DH) (JCPDS card no.99-0058). With the addition of OPC, the characteristic peak of ettringite can be noticed on at the XRD patterns.

### 3.4. X-ray Photoelectron Spectroscopy (XPS) Spectra Analysis

**Figure 5:**
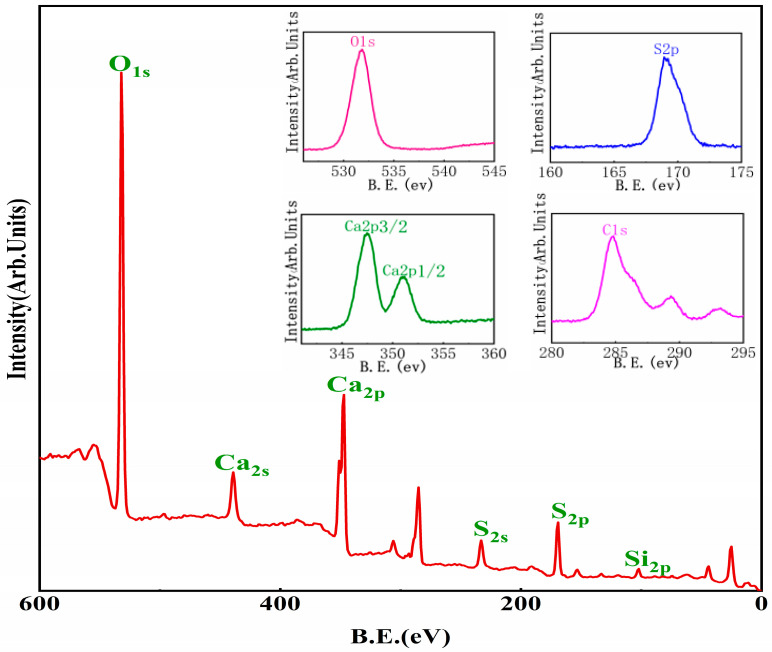
XPS spectra of HPG-E 50 (the inset is high-resolution spectra of O1s, S2p, Ca2p_1/2_, and C1s, respectively).

The XPS spectra indicates the chemical states and surface chemical composition of specimens. Figure 5 exhibits the overall spectra of the HPG-E 50, and the inserted pictures give the high-resolution spectra of O1s, S2p, Ca2p_1/2_, and C1s, respectively. The binding energy values of S2p was observed to be 169–170 eV, which could be assigned to S^4+^ of CaSO_4_. The O1s binding energy of HPG-E 50 (532.40 eV) is considered characteristic of the O^2−^ oxidation state for the SO_4_^2−^. The Ca(2p_2/3_) peak appears at 346.7 eV due to the association of calcium in C-S-H. The binding energy difference between the Ca(2p_2/3_) and Ca(2p_1/2_) peaks about 3.5 eV, it is typical in calcium sulfate, silicate, and carbonate. A chemical shift with respect to the Si(2p), Si-oxidation state for these silicate minerals occurs at around 102–103 eV.

### 3.5. Brunauer-Emmett-Teller (BET) Surface Area Analysis

**Figure 6:**
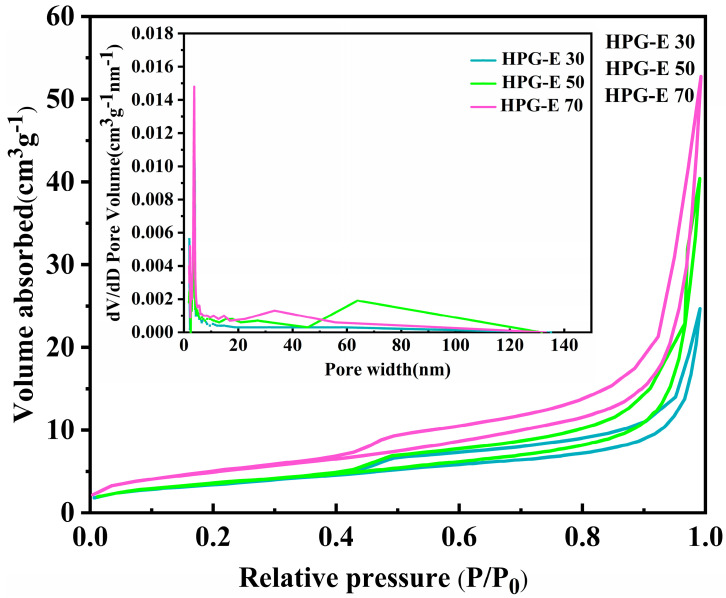
Nitrogen adsorption/desorption isotherms and pore diameter distribution (the inset is plotted using the BJH method).

Nitrogen sorption isotherms were measured, and BET surface area analysis was performed to observe the changes for HPG-E composites (Figure 6 and Table 6). With the increase of β-HPG content, the specific surface area of the HPG-E composites increased. When the content of β-HPG is 70 wt%, the specific surface area can reach 18.03 m^2^/g, indicating that the existence of β-HPG can effectively improve the specific surface area of the composites and could be prepared into lightweight building materials with respiratory function. The N_2_ sorption isotherms presented a typical type IV character with a clear H1-type hysteresis loop, which obviously suggests the formation of mesopores in the HPG-E composites. The pore size distribution further confirmed the presence of mesopores, with the maximum at around 4.2 nm in the range of 2–20 nm.

### 3.6. Waterproof Properties of HPG-E Composites

**Figure 7:**
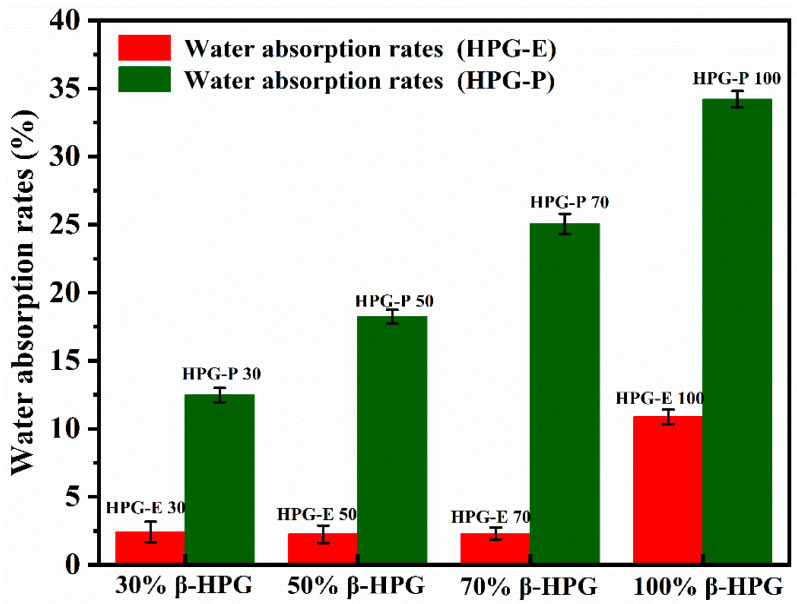
Water absorption properties of the as-prepared specimens.

**Figure 8:**
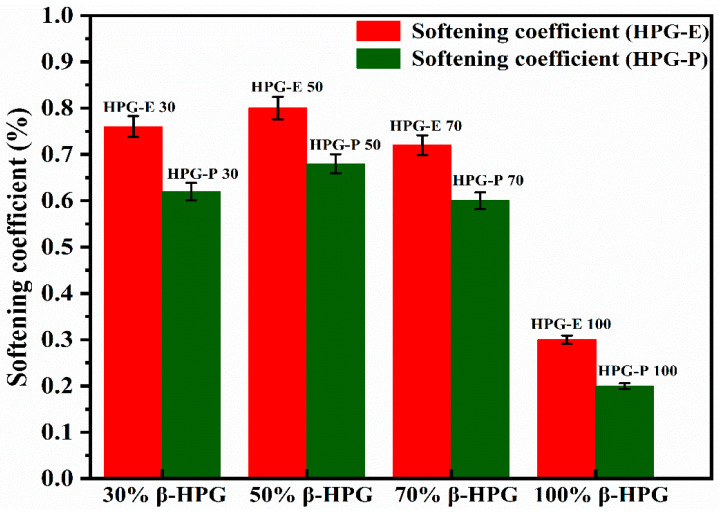
Softening coefficients of the as-prepared specimens.

**Figure 9:**
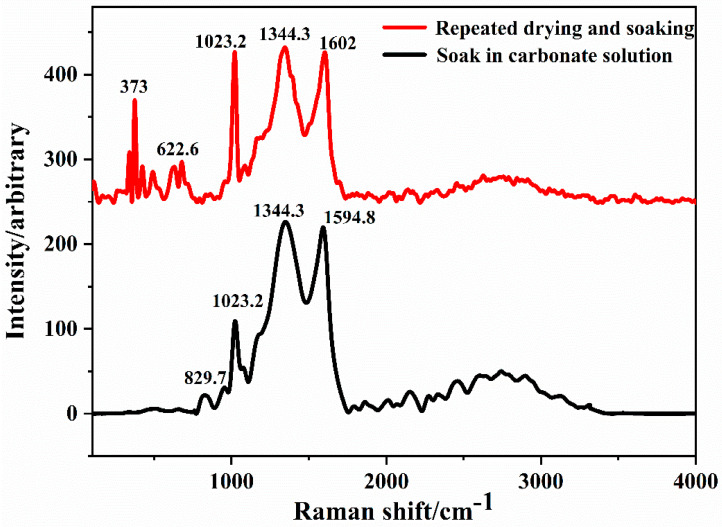
Raman spectroscopy analysis of HPG-E 50 after repeated drying and soaking 6 times, and soaked in water containing carbonate ions for 6 months, respectively.

Water absorption rate and softening coefficient are indexes to evaluate the waterproof properties of HPG-E composites. Figure 7 shows the water absorption rate of specimens with different content of β-HPG. For the HPG-P group, the water absorption rates of specimens increased significantly with the increasing content of β-HPG after being soaked in water for seven days, but the absorption rates of HPG-E composites (HPG-E 30, HPG-E 50, and HPG-E 70) were maintained at a stable low level (~2.5%). The softening coefficient was used to supplement the water-resistance of the HPG-E composites (Figure 8). When the contents of β-HPG ranged from 30% to 100%, the softening coefficients of specimens were increased by 22.58%, 17.64%, 20%, and 50%, respectively. Particularly, the softening coefficient of HPG-E 50 was about 0.78. It indicates the HPG-E composites have a better waterproof property performance compare with the HPG-P composites. To investigate thaumasite formation, specimens were repeatedly soaked and dried for six times and soaked in water containing carbonate ions for six months, respectively. Through Raman spectrum analysis, there was no thaumasite (as shown in Figure 9). There are two main peaks (990 cm^−1^ and 1076 cm^−1^) for thaumasite formation, which corresponds to sulfate and carbonate, respectively [37]. It is confirmed that the HPG-E composites can withstand the corrosion of carbonate ions.

### 3.7. The Radiation Index of HPG-E Composites

The internal exposure index (*I_Ra_* = 0.5) and external exposure index (*I_γ_* = 0.6) of HPG-E composites fully meet the requirements (*I_Ra_* ≤ 1.0, *I_γ_* ≤ 1.0) of the Chinese standard (GB 6566-2010) (Table 7).

### 3.8. Large-Scale of Bioinspired Phosphogypsum Composites

**Figure 10:**
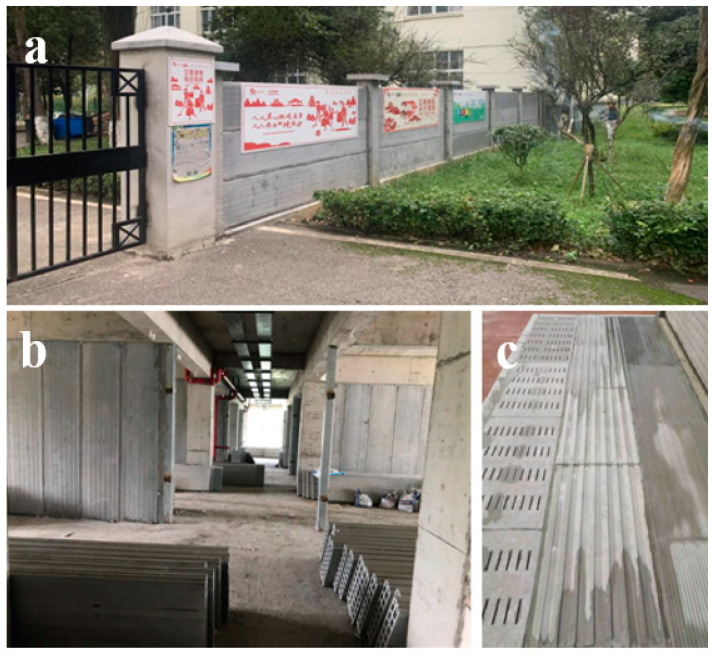
(**a**) PG exterior wall in the campus of Guizhou Institute of Technology produced from HPG-E composites; (**b**) PG interior wall panel produced from HPG-E composites; (**c**) PG pavement produced from HPG-E composites.

To achieve the practical application of HPG-E composites, we have prepared HPG-E exterior wall at the GIT campus (Figure 10a), interior wall panel (Figure 10b), and pavement (Figure 10c), which have been used in construction (Supplementary Movie S2).

### 3.9. Simulation Results of Reactive Extrusion

**Figure 11:**
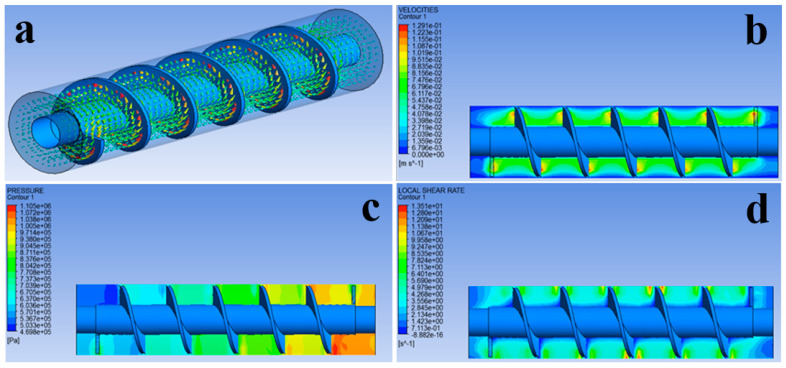
Simulation of screw extrusion. (**a**) 3D flow diagram; (**b**) velocity program; (**c**) pressure program; (**d**) shear rate program of HPG-E composites in the reactive extrusion process.

To prove the mechanism of reactive extrusion, the screw extrusion process is simulated by using POLYFLOW software. The rotating single screw extruder in this study consisted of a flow channel and single screw elements. We define ‘screw’ as the moving part and the flow channel as the domain. Meshing is carried out with the preprocessor software GAMBIT. The mesh superposition technique is used to put the flow channel and screw together without remeching for the periodical changes. The boundary condition for the input was set as inflow and output as outflow. The tube wall and surface of the screw are free surfaces, which means the normal and tangential forces are zero. The material of the screw is 38CrMoAl and the rotation speed of the screw set as 35 rpm. The AFM direct solver is used as the solving algorithm. As shown in Figure 11a,b, under the screw extrusion, the material transportation process included shear, friction and extrusion. It formed a complex flow field near the screw. Through the analysis of the pressure program (Figure 11c), it can be seen that the overall pressure trend gradually increased with the screw rotation during the extrusion process. Under the action of viscosity, the drag surface pulled the material forward. The pressure at the outlet increased since more materials have been pushed to the end. The screw requires more force to push the materials to the outlet and form the desired shapes. Hence, the highest pressure appeared at the outlet of the process. Figure 11d shows the distribution of shear rate of the flow in the screw. As expected, the highest shear rate occurred between the tip of the screw and the barrel. In the same ways as the velocity contour shown in Figure 11b, the velocity of the flow was at its maximum at these locations since the gaps were narrowest. The shear rate in the screw was affected by screw diameter, screw rotation speed, and the channel depth. In this case, the screw diameter and the screw speed remained the same through the process. Channel depth would be the factor that influence the shear rate. The shear rates increased with the length of the channel depth. From the point of view of crystal growth kinetics, growth driving force is an important dynamic factor affecting the crystal’s directional growth. During the hydration process of calcium sulfate hemihydrate crystal, it formed multilayer overlapping microstructure with calcium silicate hydrate under the action of pressure flow in screw extruder. Under this pressure trend, the crystal can perform directional growth. The exact pressure range is not yet known. It requires further research and exploration.

## 4. Conclusions

In conclusion, this work provides a simple and efficient strategy for preparing large-scale bioinspired phosphogypsum composites with high mechanical and waterproof properties. The results of this study can be summarized as follows:

(1) The screw extruder can improve the mechanical strength and waterproof properties of β-HPG building materials without any special chemical admixtures and cements. After curing for seven days, with more than 50 wt% of β-HPG, the compressive strength and softening coefficient were 24.98 MPa and 0.78, increasing by 110.0% and 20.0%, respectively, compared to the pouring method.

(2) The pressure increased along the length of the screw with the screw rotation. During the extrusion process, the calcium sulfate dihydrate crystals can perform directional growth under the action of pressure. However, further research and exploration are required to find the exact pressure range and the exact rotation condition in the future. Specifically, we should further simplify the synthesis process with the goal of scaling up to even larger throughputs.

(3) The multi-level cross-laminated microstructure can effectively resist the external impact force; this kind of architecture is very similar to the nacre’s microstructure. However, it needs to be pointed out that β-HPG still contains impurities. These impurities affect the calcium sulfate can not fully achieve directional growth of two-dimensional crystalline in the extrusion process. In the coming research, we are going to perform deep removal of impurities and purification of phosphogypsum.

## Figures and Tables

**Table 1:** The main chemical composition of β-HPG (by weight, wt%).

No. *	SO_3_	CaO	SiO_2_	P_2_O_5_	Fe_2_O_3_	Al_2_O_3_	MgO	TiO_2_	F	Loss
1	51.77	40.21	2.55	0.42	0.45	0.53	0.39	0.09	0.56	2.16
2	49.80	41.73	1.62	0.72	0.62	0.53	0.51	0.06	0.75	2.22
3	51.40	39.12	3.15	0.65	0.56	0.96	0.62	0.08	0.66	2.03

* Source of β-HPG: 1. Kingenta Ecological Engineering Group Co., Ltd. 2. Guizhou Kaidi green building materials Co., Ltd. 3. Guizhou Honghai gypsum powder industry Co., Ltd.

**Table 2:** Chemical and Bogue composition of of OPC (by weight, wt%).

Chemical Composition											Bogue Composition			
SiO_2_	Al_2_O_3_	Fe_2_O_3_	CaO	MgO	K_2_O	Na_2_O	SO_3_	TiO_2_	LOI	Others	C_3_S	C_2_S	C_3_A	C_4_AF
21.86	4.45	2.35	63.51	1.67	0.55	0.26	2.91	0.11	1.89	0.44	30	45	6	12

**Table 3:** The formulation of the HPG-P composites.

No.	Mix Proportions (wt%)			
	β-HPG	OPC	Polycarboxylate Superplasticizer	Retarder
HPG-P 10	10	90	0.2	0.2
HPG-P 30	30	70	0.2	0.2
HPG-P 50	50	50	0.2	0.2
HPG-P 70	70	30	0.2	0.2
HPG-P 100	100	0	0.2	0.2

**Table 4:** The formulation of the HPG-E composites.

No.	Mix Proportions (wt%)				
	β-HPG	OPC	Hypromellose	Polycarboxylate Superplasticizer	Retarder
HPG-P 10	10	90	0.3	0.2	0.2
HPG-P 30	30	70	0.3	0.2	0.2
HPG-P 50	50	50	0.3	0.2	0.2
HPG-P 70	70	30	0.3	0.2	0.2
HPG-P 100	100	0	0.3	0.2	0.2

**Table 5:** The specific parameters of the single-screw extruder.

Parameter	Value
Screw tip diameter (*D*)	100 mm
Screw root diameter (*d*)	42 mm
Screw length (*L*)	370 mm
Chanel depth (*h*)	29 mm
Pitch of thread (*P*)	80 mm
Wall thickness (*t*)	6 mm
Screw rotation speed (*v*)	30 rpm

**Table 6:** N_2_ gas adsorption-desorption analyses of the as-prepared samples.

Samples	BET Surface Area (m^2^g^−1^)	Pore Volume ^a^ (cm^3^g^−1^)	Pore Size ^b^ (nm)
HPG-E 30	12.48	0.04	11.82
HPG-E 50	15.25	0.06	16.02
HPG-E 70	18.03	0.08	16.95
HPG-E 100	27.74	0.09	12.42

^a^ The pore volume was investigated from the volume of N_2_ adsorbed at ~P/P_0_ = 0.99. ^b^ The average pore size was calculated using the BET method.

**Table 7:** The content of radionuclide and specific radioactivity for the HPG-E composites.

	Demand for Standard	Test Result
Radionuclide	*I_Ra_* ≤ 1.0, *I_γ_* ≤ 1.0	*I_Ra_* = 0.5, *I_γ_* = 0.6
Specific radioactivity	/	*C_Ra_* = 107.88 Bq/Kg
		*C_Th_* = 515.16 Bq/Kg
		*C_K_* = 400.30 Bq/Kg

## Data Availability

The data that support the findings of this study are available from the corresponding author upon reasonable request.

## Supplementary Materials

The following are available online at https://www.mdpi.com/article/10.3390/ma14195601/s1.
